# Oil Recovery Improvements Based on Pickering Emulsions Stabilized by Cellulose Nanoparticles and Their Underlying Mechanisms: A Review

**DOI:** 10.1021/acsomega.4c08428

**Published:** 2025-01-20

**Authors:** Roberta T. Pinto, Karla S. Feu, Cleocir J. Dalmaschio, Andreas Nascimento, Valdemar Lacerda

**Affiliations:** † LabPetro - Department of Chemistry, Center for Exact Sciences (CCE), Federal University of Espírito Santo (UFES), Vitória, ES 29075-910, Brazil; ‡ Institute of Mechanical Engineering, Federal University of Itajuba (UNIFEI), Itajuba, MG 37500-903, Brazil

## Abstract

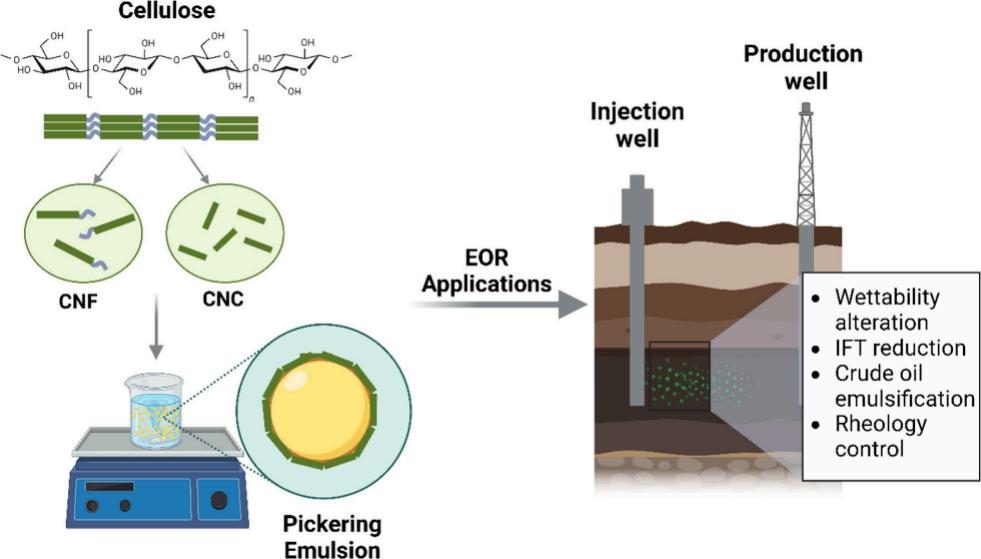

The use of nanocellulose (NC)-based Pickering emulsions represents an advancement in chemically enhanced oil recovery (cEOR) methods. The main challenge of cEOR is to develop stable and efficient fluids for applications under reservoir conditions. Pickering emulsions have emerged as a possible solution for stabilizing chemical injection fluids. These emulsions are stabilized by solid particles instead of surfactants and have been the focus of research over the past decade because of their high stability. Although these emulsions present promising solutions, most research has focused on nonbiodegradable inorganic particles, raising concerns about their environmental impact. In this context, nanocellulose (NC) has emerged as an innovative and sustainable alternative due to its biodegradability, abundance, and unique surface chemistry. This contribution presents an exploratory literature review on the use of Pickering emulsions, focusing on nanocellulose in the context of enhanced oil recovery (EOR) as an alternative for fluid stabilization under reservoir conditions. The main mechanisms of oil recovery, such as interfacial tension reduction, *in situ* crude oil emulsification, capillary disjunction, pressure, and fluid rheological behavior, are discussed. This Review highlights the great potential of nanocellulose-based Pickering emulsions to make EOR processes more sustainable and emphasizes the need for further studies to understand the mechanisms involved. A total of 176 scientific articles were analyzed and evaluated to provide insights and contribute to the advancement of cEOR, in addition to addressing the challenges encountered.

## 1 Introduction

Oil has been the main global source of energy and raw materials in the last century, with a growing demand due to the high standard of living and rapid development of the industrial sector. Furthermore, while global energy requirements continue to rise, mature oil fields face declining production rates, coupled with diminishing discoveries of new viable reserves. In this context, the field of enhanced oil recovery (EOR) research was established, and researchers are focused on developing new technological methods to improve oil recovery efficiency, increase recovery factors, and ensure economic viability in mature petroleum fields.^1^

Petroleum can be defined as a complex mixture composed of different cyclic, aliphatic, and aromatic hydrocarbons that can be in solid (asphalt and bitumen), liquid (oil), or gaseous (natural gas) states. These fluids are found underground, occupying the pore space of sedimentary rocks, called reservoir rocks. In addition, the presence of water and contaminants such as metals, sulfides, and carbon dioxide is common in the petroleum found in these reservoirs.^2,3^

In addition to long periods of exploration, the oil industry faces challenges during the oil production stage, where only 30–40% of the original oil *in place* (OOIP) is successfully recovered during extraction. Approximately 60%–70% of the OOIP remains trapped in the pores of the reservoir rock.^4^ The oil remains in the reservoir due to properties such as high viscosity, high interfacial tension, and wettability of the rock to oil, which hinder oil mobility. To mitigate this decline in production, enhanced oil recovery (EOR) techniques are applied with the goal of altering the physicochemical characteristics of rock and oil, thereby increasing oil recovery and extending reservoir longevity. EOR methods are divided into thermal, miscible, chemical, and microbiological methods.^2,5^

Chemical enhanced oil recovery (cEOR) is an EOR technique in which chemicals, such as polymer solutions, surfactants, and alkaline agents, are injected into a reservoir. These compounds counteract the mechanisms that cause oil retention in the reservoir rock. Polymer solutions improve the mobility ratio by increasing the viscosity of the injected water and reducing the formation of preferential channels (fingering) during the injection process. Surfactants reduce the interfacial tension (IFT) between oil and water and form *in situ* emulsions, making it easier to displace oil trapped in the pores. Alkaline agents are used to alter the wettability of rock and react with the naphthenic acids present in the oil to form surfactants *in situ*, which promote oil emulsification and enhance its mobility.^6^

However, depending on the reservoir conditions, some chemical compounds have limited applications. For example, polymers degrade at high temperatures and clog the pores of the rock. Surfactants have an unfavorable mobility ratio and are lost because of their adsorption and retention in the rock. On the other hand, alkaline agents cause corrosion problems in pipes and contaminate the oil layer, leading to encrustation. These events cause significant damage and negatively impact reservoir exploitation.^7,8^

Some oilfields have applied flood tests using ASP solutions (alkali/surfactant/polymer), and the synergy between the compounds has demonstrated greater efficiency than separate use.^4^ During such tests, researchers noticed that the efficiency of oil recovery in ASP floods was minimal in the absence of emulsion formation. This fact demonstrated the importance of forming an emulsion *in situ* between the crude oil and the injected solution.^1,8^ Several studies have replaced conventional flood fluids with emulsions, with the aim of greater efficiency. However, the low stability of the emulsion resulted in low oil recovery, making the process economically unviable in the long term.^9,10^

Recently, Pickering emulsions have emerged as attractive chemical flooding fluids because of their cost-effectiveness and high stability even under high temperature, pressure, and salinity. Such emulsions have the potential to improve EOR processes and enhance oil production if properly implemented.^8,11,12^ Furthermore, biobased polymers are more eco-friendly than synthetic polymers.

Pickering emulsions are surfactant-free and stabilized by particles or nanoparticles (NPs) that adsorb at the interface of two immiscible fluids, producing highly stable ones.^13^ These emulsions have several advantages over conventional emulsions stabilized by surfactants.^14^ The stabilization efficiency is based on their unique properties, including small particle size, large surface area, surface charge, composition and architecture.^15−19^

Different types of organic or inorganic NPs can be used as stabilizers for Pickering emulsions.^20^ The most common inorganic NPs include silica NPs,^21,22^ titanium dioxide,^23^ zinc oxide,^24^ and graphene oxide.^25^ The most common organic NPs are starch,^26,27^ chitosan,^28^ and cellulose.^29,30^ Furthermore, mixed NP-stabilized Pickering emulsions,^31^ such as Janus NPs, which have unique properties, have attracted the attention of researchers.^32^

Most of the research related to EOR techniques involves the use of nonrenewable and nonbiodegradable NPs, which can have adverse effects on the environment. In this context, researchers are increasingly developing Pickering emulsions using organic and biodegradable NPs. Nanocellulose (NC) is an NP produced from cellulose, the most abundant biodegradable and renewable polymer in the world, with an annual worldwide production of 7.5 × 10^10^ tons.^33^ Cellulose originates from plants, algae, animals, and microorganisms. It is a versatile, eco-friendly material with unique properties, such as a large surface area, easy chemical modification, and excellent colloidal and interfacial properties.^6,34,35^

Different types of nanomaterials can be derived from cellulose such as cellulose nanofibers (CNFs) and cellulose nanocrystals (CNCs). Li et al.^35^ studied the amphiphilic properties of CNCs and their application in stabilizing emulsions at different pH values, ionic strengths, and temperatures. Heggset et al.^36^ reported that the thermal stability of CNFs and CNCs is superior to that of xanthan gum, the most commonly used biopolymer in EOR. These characteristics are promising for EOR applications because the temperature, pH, salinity, and pressure conditions vary among oil reservoirs.

Although there has been remarkable progress related to the application of NC in different sectors of the oil industry, studies dedicated to its application as a stabilizer in Pickering emulsions for EOR projects are scarce.^37,38^ Thus, the objective of this review article is to summarize the literature concerning cellulose nanoparticles as stabilizers of Pickering emulsions with potential applications in enhanced oil recovery between 2000 and 2023. With technological advancements, nanotechnology can make the industry more sustainable and reduce the carbon footprint compared to conventional production methods. These factors contribute to making the upstream oil and gas sector more efficient in recovery processes. In conclusion, we aim to inspire further research on the development of Pickering emulsions formed from NPs for oil recovery.

An exploratory literature review was conducted in the Scopus and Web of Science databases using the terms “EOR” + “Nanocellulose” and “EOR” + “Pickering emulsion” between 2000 and 2023 to identify publications from the main countries contributing to the field. The survey was conducted on November 5, 2024, and a total of 286 scientific articles were identified via search terms in the titles, abstracts, and keywords. The leading countries in publications on the subject are China, Iran, the USA, and Norway. Among these, China stands out with 163 articles, representing 57% of the total publications.

In 2014, worldwide daily oil production using the cEOR method accounted for 1.51%. However, China had a significantly larger share, with 17% in 2014 and 54.72% in 2016, reflecting China’s increased investment in cEOR methods. These data highlight the importance of China’s investment in cEOR methods, especially given the presence of mature fields in the high-water cutting phase. These fields require enhanced recovery techniques to increase production and profitability.^39^

Exploratory literature search data were exported in BibTeX format and analyzed in RStudio software with the bibliometrix tool.^40^ This bibliometric analysis makes it possible to understand the patterns of publication and collaboration between countries in the areas of EOR, nanocellulose, and Pickering’s emulsions.

## 2 Pickering Emulsions

**Figure 1:**
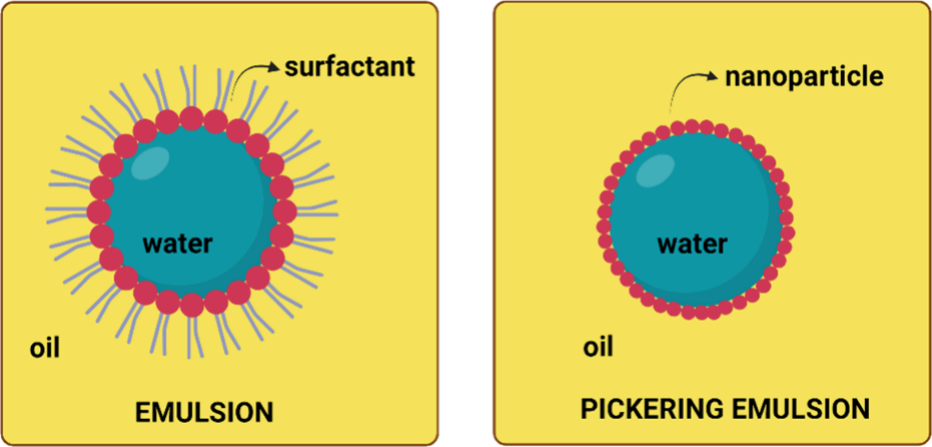
Graphical representation of a classic emulsion (based on a surfactant) and a Pickering emulsion. The nanoparticles are adsorbed at the oil–water interface and stabilize the droplets in place of the surfactant molecules. Created with BioRender.com.

Emulsions are systems formed by two immiscible liquids, typically consisting of a continuous phase and a dispersed phase. The droplets of one liquid are dispersed in the other with the help of stabilizing agents such as surfactants or solid particles (Figure 1).^41−43^

An emulsion is a thermodynamically unstable system due to the high interfacial energy between the immiscible phases. Conventional emulsions are stabilized by chemical surfactants, which adsorb at the interface between liquids and reduce interfacial tension.^41^ However, Ramsden^44^ and Pickering^45^ independently reported that solid colloidal particles, such as clays and copper sulfate with lime, could adsorb at the interface between two liquids. This adsorption led to the formation of stable emulsions. Nevertheless, interest in these emulsions was limited due to the materials and technologies available at the time. With advancements in science and new technologies, it has become possible to design particles with adjustable characteristics for use as efficient stabilizers of Pickering emulsions.^35,41^ Faced with this scenario, interest in this system has increased in the past decade as a result of research and applications in various fields, such as food,^46^ drug delivery,^47^ cosmetics,^48^ and oil recovery.^49^ With the growth in the number of publications and patents in the field of Pickering emulsions, a simultaneous increase in commercial interest has been observed.^50^

The solid particles irreversibly adsorb at the fluid–fluid interface, forming a film that prevents the coalescence of the droplets.^51^ This fact confers high stability to this type of emulsion compared with conventional emulsions stabilized by surfactants. The stability of Pickering emulsions can be influenced by multiple factors, including the wettability, morphology, charge, concentration, and size of solid particles. External conditions, such as the salt concentration, pH, particle adsorption kinetics at the interface, and ratio of the oil phase to the water phase, also play crucial roles.^15,52,53^

### 2.1 Wettability and Adsorption of Nanoparticles at Interfaces

**Figure 2:**
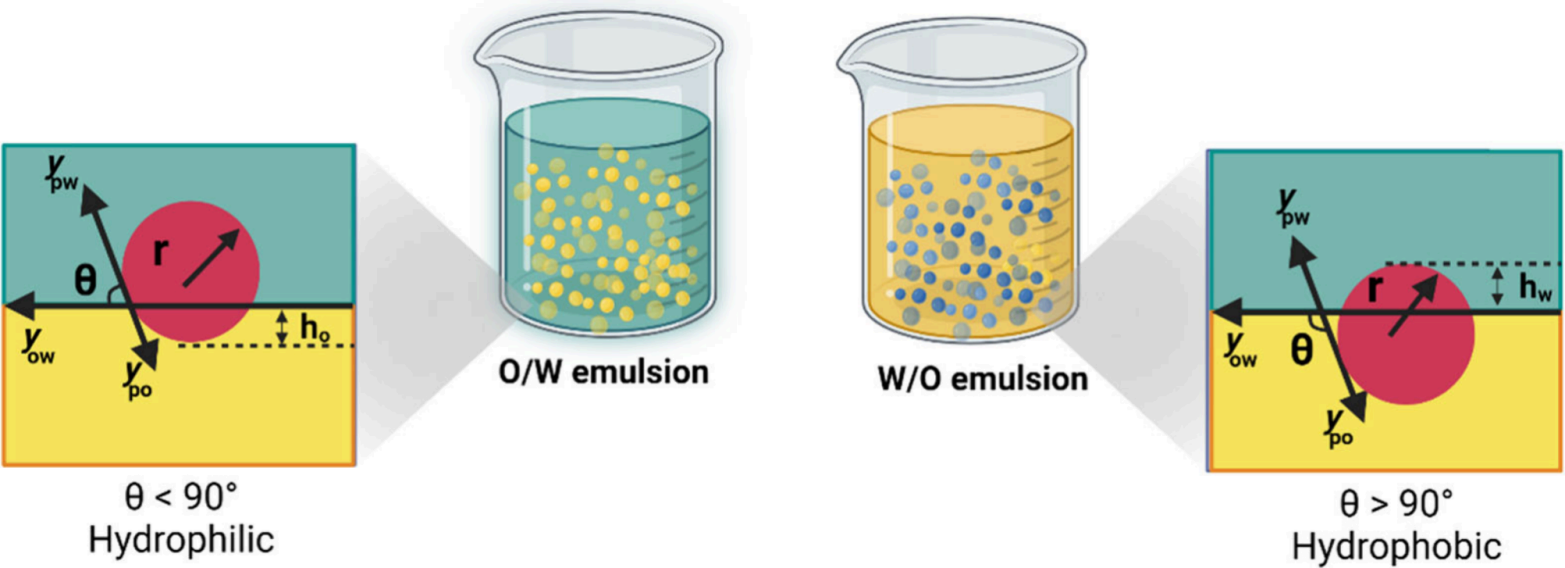
Wettability of solid particles and contact angle leading to the formation of O/W or W/O emulsions.^61,62^ Created with BioRender.com.

Pickering emulsions can be formed in different types, such as oil-in-water (O/W), water-in-oil (W/O), or multiple emulsions, with the type being determined by the wettability of the solid particles.^54^ Wettability determines the tendency of a fluid to adhere to a solid surface in the presence of other immiscible fluids.^2^ Particle wettability is a determining factor for the formulation of stable Pickering emulsions and is characterized by the three-phase contact angle (θ) between the aqueous phase, the oil phase and the particles (Figure 2).^55^ Particles with contact angles between 15° < θ < 90° stabilize O/W emulsions, whereas particles with contact angles between 90° < θ < 165° stabilize W/O emulsions.^56,57^ Particles with θ equal to or close to 90° can form double emulsions due to their ability to adsorb at the O/W and W/O interfaces.^58^ The contact angles of water (θ_*w*_) and oil (θ_*o*_) are defined by Young’s equation (eq 1):^59^
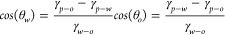
1where *γ*_*p–o*_, *γ*_*p–w*_, and *γ*_*o–w*_ are the particle–oil, particle–water and oil–water interfacial tensions, respectively. Xiao et al.^60^ studied ways to change the wettability of particles to increase the possibility of industrial applications. The authors described methods of changing the chemical structure or inserting functional groups on the surfaces of the particles to obtain the desired wettability.

According to Peito et al.^15^ the polarity and viscosity of the oil phase are parameters that influence the contact angle and can affect the droplet size, type and stabilization of the emulsion. Binks and Lumsdon^63^ reported that silica nanoparticles with intermediate wettability stabilized O/W emulsions when used with nonpolar oils and W/O emulsions when used with polar oils due to their adaptability to the surrounding phase. Thus, the silica NPs demonstrated hydrophobic characteristics with polar oils and hydrophilic characteristics with nonpolar oils. The stability of Pickering emulsions relies on particle adsorption at the oil–water interface, which is influenced by the partial wettability of the particles by the aqueous and oil phases.^15^ This is related to the interfacial energies of the three interfaces: particle–water (*γ*_*p*__–*w*_), particle–oil (*γ*_*p*__–*o*_) and oil–water (*γ*_*o*__–*w*_).^53^

The high stability of Pickering emulsions is due to the irreversible adsorption of the particles at the oil–water interface. High energy is required to remove the particles, which is up to 10^8^ times greater than the thermal energy.^55,64^ The contact angle, radius, and interfacial tension determine the adsorption energy, with the adsorption energy being highest when θ is 90°.^15^

That is, when θ is 90°, the adsorption energy is at its maximum and exceeds the thermal energy. From an energetic standpoint, the particles are considered to be irreversibly adsorbed at the interface. This adsorption, which results in a dense monolayer of particles at the interface, does not act by reducing the interfacial tension but instead forms a rigid interfacial mechanical barrier. By forming a dense monolayer, the structure prevents the rearrangement necessary for coalescence to occur, ensuring the high stability of the Pickering emulsion.^65,66^

The adsorption free energy (*ΔG*_*d*_) is defined as the energy necessary to remove a solid particle with a spherical shape of radius *r* and a three-phase contact angle with interfacial tension from the oil–water interface (*γ*_*o*__–*w*_), as described in eq 2.^59,67^
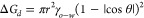
2

The partial wettability of a particle in an oil phase by water requires a water adsorption energy () with a positive value and a negative water scattering coefficient (), according to eqs 3 and 4:^53^
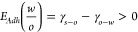
3
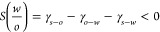
4

The same occurs with the oil wettability of the particle within the aqueous phase, where the positive adhesion energy () and the negative scattering coefficient () of the oil are observed as described in eqs 5 and 6:^53^
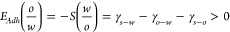
5
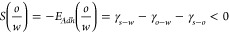
6

A particle with a completely hydrophilic surface is wetted by water, so its adsorption at the interface does not occur, as the particles are dispersed in the aqueous phase. Likewise, particles with a completely hydrophobic surface are wetted by oil, consequently dispersing in the oil phase and not adsorbing at the interface.^63^ Under conditions of partial wettability, the free energy of adsorption is high and is related to the values of interfacial tension and particle size, given by eqs 7 and 8 for spherical particles of radius *R*. Maximum adsorption is reached when θ = 90°, at which point the highest emulsion stability is achieved.^53^
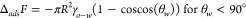
7
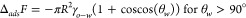
8

### 2.2 Size and Shape of the Nanoparticles

Particle size directly influences the stability of the Pickering emulsions. To obtain stable emulsions, the particles must be an order of magnitude smaller in diameter than the smallest droplets present in the emulsion.^59,67^ The size of the particles is described as being at the nanoscale (nanoparticles) or microscale (microparticles).^15^

According to Tsabet and Fradette,^68^ the emulsion stabilization mechanism can be characterized in three steps: (i) the particle approaches and reaches the interface, (ii) the particle adsorbs at the interface, and (iii) the adsorbed particles stabilize the emulsion, forming a network. When the particle size increases, the repulsive and electrostatic forces of the double layer also increase, which impairs the particle’s ability to approach the interface. In addition, the adsorption time increases with increasing particle size, making the second step more difficult since adsorption is a function of the particle/oil affinity, which is related to the particle contact angle. Smaller particles exhibit faster adsorption kinetics, resulting in more stable emulsions. This is due to the absence of an adsorption barrier, leading to more efficient packing at the oil–water interface.^41^

**Figure 3:**
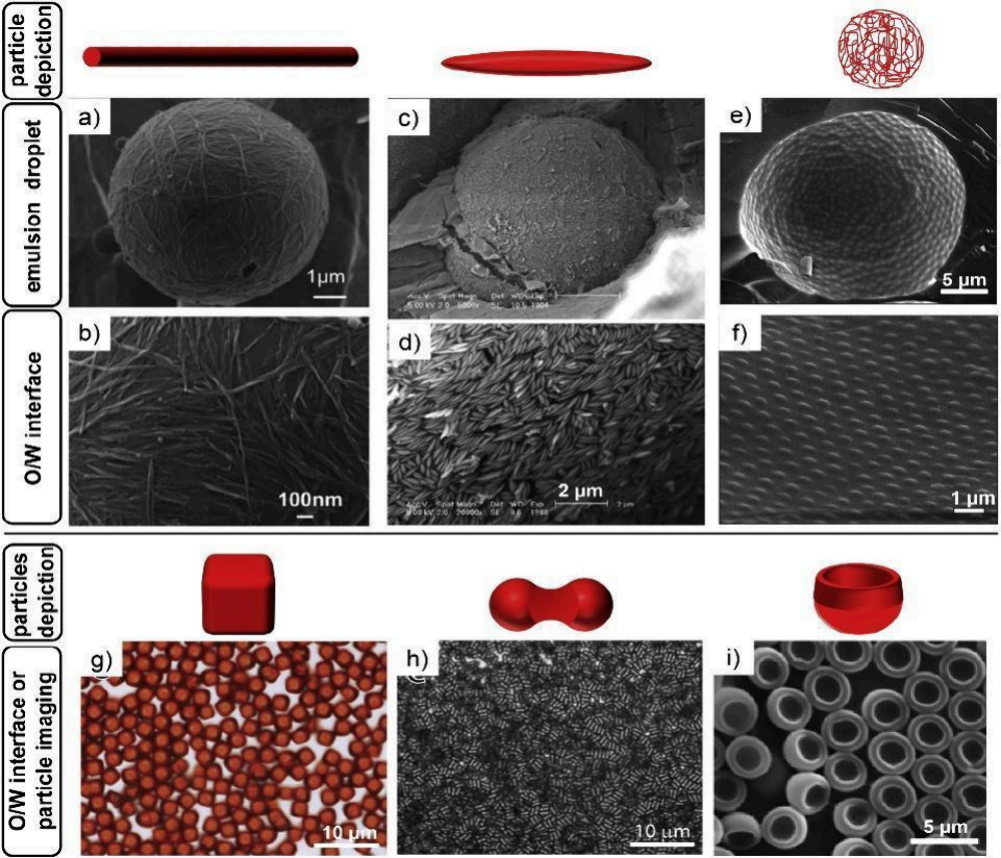
a) and b) SEM images of polymerized styrene–water emulsions stabilized by bacterial cellulose nanocrystals, c) and d) Cryo-SEM images of a drop of water covered with ellipsoids, e) and f) Cryo-SEM images of drops of dodecane coated with microgels, g) array of cubic particles at the oil–water interface, h) peanuts mounted at the oil–water interface in interdigitating stacks, and (i) SEM images of microbowls but not at the O/W interface. Reprinted with permission from *J. Controlled Release***2019**, *309*, 302–332. 10.1016/J.JCONREL.2019.07.003.^59^ Copyright 2019, Elsevier.

The shape of the particles and their adsorption and organization at the interface influence the stability of the emulsion.^69^ Initially, Pickering emulsions were developed using spherical particles; however, particles with nonspherical shapes, such as ellipsoids,^70^ rods,^71^ fibers, cubes, microgels, microbows, Janus, peanuts, and others (Figure 3),^59^ have been used in formulations of stable W/O,^70^ O/W^71^ and multiple emulsions.^72^

Adsorption energy equations are not applicable to these nonspherical particles, and their emulsion stabilization mechanisms remain poorly understood.^59^ Since the equations take into account the contact angle of spherical particles, it is not possible to apply these equations to particles with other morphologies. Additionally, it is necessary to consider the particle orientation at the interface and at least two characteristic sizes, which makes these equations inadequate for determining adsorption energies.^73^ Some characteristics, such as flexibility, conformation, and the creation of a particle network at the interface, favor greater coverage around the drop and increase the stability of the emulsion.^15^

### 2.3 Particle Concentration

**Figure 4:**
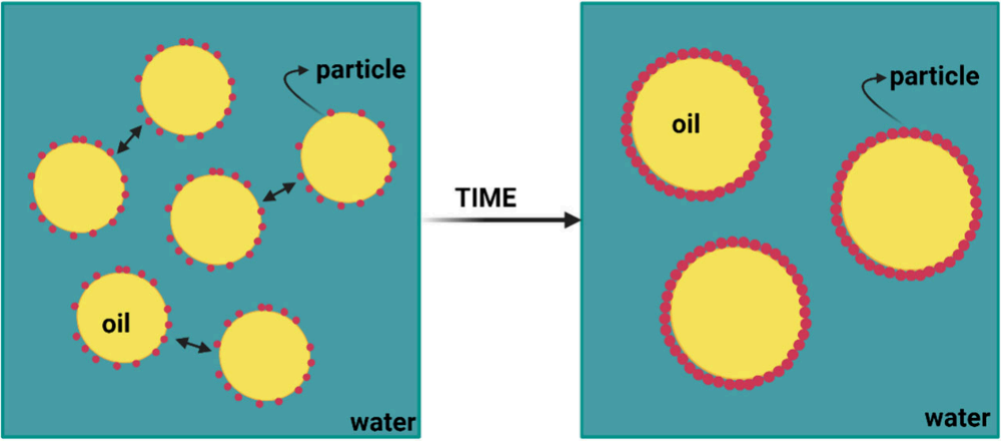
Illustration of the bounded coalescence theory; the double arrows outline the approaching droplets. Created with BioRender.com.

In addition to particle size and shape, another factor that influences the stability of Pickering emulsions is particle concentration.^54,74^ Research reports a correlation between the droplet size and particle concentration. Concentrations can be classified as low, intermediate, or high in relation to the interfacial area generated during the emulsification process.^75^ At intermediate concentrations, the number of particles is slightly lower in relation to the interfacial area, leading to droplet coalescence until a sufficient number of particles is available to cover the interface. This phenomenon is known as limited coalescence, meaning that at intermediate concentrations partial coverage of the newly formed droplets occurs. The number of particles does not cover the entire interface but is enough to prevent uncontrolled coalescence. The droplets subsequently coalesce, allowing the particles to redistribute and form a dense layer at the interface that prevents continuous coalescence (Figure 4).^75−77^

Typically, emulsion stability increases with a higher particle concentration; at high concentrations, rapid stabilization of the formed droplets may occur, leading to heterogeneous droplet sizes or excess particles forming a network in the continuous phase. This network can stabilize the droplets and enhance stability by preventing the coalescence mechanism. Additionally, at high concentrations, phase inversion may occur, transitioning from an O/W emulsion to a W/O emulsion and vice versa or to multiple emulsions.^59^ Emulsion stability does not occur at low concentrations, as there are not enough particles to cover the interfacial region, resulting in droplet coalescence.^19,69,73^ Binks et al.^77^ reported that phase inversion due to an increase in the NP concentration depends on their hydrophobicity and the phase in which they were initially dispersed. This study evaluated pyrogenic silica particles with varying hydrophobicity, where phase inversion from O/W to W/O occurred only in systems with particles of intermediate hydrophobicity (57% and 71% SiOH groups on the surface) when dispersed in oil. At high concentrations, aggregates are formed and stabilized by hydrogen bonds between silanol groups, which increase the hydrophobicity of the particles, causing them to accumulate at the interface with a greater tendency to stabilize W/O emulsions.

Frelichowska et al.^78^ utilized silica nanoparticles to investigate the effect of their concentration on the stability of O/W emulsions. Formulations with concentrations less than 1% by weight did not demonstrate stability. Furthermore, the authors reported an inverse relationship between the droplet size and the concentration of silica NPs. The conclusion is that the stability of the emulsion increases with increasing concentration since a decrease in the droplet size implies a larger interfacial area.

### 2.4 Particle Charge, pH and Ionic Strength

The magnitude of the surface charge of the particles is an important parameter for determining the stability of Pickering emulsions. The surface charge can be obtained by the zeta potential technique (ζ). If the value is ζ > ±30 mV, electrostatic repulsion is the dominant interparticle interaction, whereas in emulsions with values between −30 mV < ζ < 30 mV, the dominant interaction is van der Waals interactions, which are attractive forces. Thus, high values of ζ impair the adsorption of particles at the interface due to the high repulsion. On the other hand, the reduction in ζ favors the aggregation of the particles and strengthens the network between them, providing greater stabilization of the droplets in the emulsion.^41,79,80^

When particles exhibit a very high zeta potential, electrostatic repulsion prevents their adsorption at the interface. However, by reducing the zeta potential to values near zero, adsorption becomes more efficient. Furthermore, in regions of low surface charge, particle aggregation occurs, forming an interfacial network within the continuous phase of the emulsion, which enhances droplet stabilization. However, it is important to monitor the surface charge of the particles during the preparation of Pickering emulsions, as a very low zeta potential can induce droplet aggregation in the emulsion.^81^

Variations in salinity and pH can change the surface charge and hydrophobicity and, consequently, the wettability of particles that have groups on their surface that can be ionized. These parameters can modify the interactions from attractive to repulsive interactions, which allows the modulation of particle adsorption and the type of emulsion.^80^ Ren et al.^82^ used hydrophilic aminosilica NPs with pH-responsive wettability by dynamic covalent bonding with hydrophobic benzaldehyde molecules. At pH 7.8, the NPs bound to benzaldehyde were partially hydrophobic, stabilizing the Pickering O/W emulsions. At pH 3.5, the bond with benzaldehyde is broken and the NPs become highly hydrophilic, resulting in phase separation of the Pickering emulsion.

According to Kalashnikova et al.^83^ cellulose NPs, which are used as Pickering emulsifiers, do not stabilize emulsions when their surface charge density is above 0.03 charge/nm. However, these conditions can be improved by the addition of salt, which screens electrostatic forces and results in the formation of stable emulsions with highly charged cellulose NPs. Tang et al.^25^ used polymer-grafted cellulose NPs and reported that the addition of NaCl reduced the coalescence process, inhibited cream formation and led to the formation of more stable emulsions. At low concentrations of NaCl, the ζ value was greater than 30 mV, and the aggregation of the NPs was reduced because of the high electrostatic repulsion. At higher NaCl concentrations, ζ is reduced, promoting the aggregation of NPs, with van der Waals attractive forces dominating. In conclusion, the addition of NaCl at low concentrations strengthens the network of cellulose nanoparticles in the continuous phase due to the increase in electrostatic repulsion, which makes their aggregation difficult, resulting in more stable emulsions.

### 2.5 Relationship between the Oil Phase and Aqueous Phase

The ratio between the aqueous and oil phases is a parameter that affects the size of the droplets and can change the type of emulsion formed.^64^ When the dispersed phase increases, the number of particles is not enough to stabilize the emulsion, as the interfacial area also increases; in addition, this increase can cause an inversion of the type of emulsion.^57,59^ Tang et al.^25^ used silver NPs and graphene oxide in Pickering emulsions with a 1:1 oil phase/aqueous phase ratio. The formed emulsions were stable for months and were of multiple A/O/A and O/A/O/A types, and the authors attributed this phenomenon to the amphiphilic properties of the silver nanoparticles and graphene oxide. When the ratio between the oil phase and aqueous phase was 2:1, the emulsion separated into two phases, demonstrating that an increase in the dispersed phase caused instability in the emulsion. Moreover, at an oil-to-aqueous-phase ratio of 1:2, the multiple emulsion droplets disappeared because of an increase in the continuous phase.

Wettability is another parameter that can change depending on the phase in which the particles are dispersed and the polarity of the oil phase.^15,64^ The nature of the oil directly influences the interfacial tension of the system and, consequently, the three-phase contact angle. Thickett and Zetterlund^84^ synthesized two-dimensional graphene oxide (2DGO) NPs to investigate the hypothesis that the amphiphilic characteristics of the NPs are influenced by the polarity of the oil phase. The authors reported that the stabilization energy of NP adsorption was greater for nonpolar oils, which formed more stable emulsions than did polar oils. This behavior occurs because by increasing the polarity of the oil the interfacial tension also increases until, at a given moment, the adsorption of the NPs at the interface is not thermodynamically favorable, promoting instability of the system.

## 3 Cellulose and Cellulose-Based Nanoparticles

Environmental concerns promote market influence to produce “clean label” and “green” products. Within the field of Pickering emulsions, these influences have motivated researchers to develop biomass-based particles made from materials derived from or isolated from plants or microorganisms.^50^ Cellulose is the most abundant biodegradable polymer in the biosphere and has been extensively applied to the production of nanoparticles. It is obtained mainly from plants (wood, cotton, plant residues and algae),^61,85^ but it can also be found in species of marine animals^86^ and microorganisms.^87^ Lignocellulosic biomass consists of cellulose (40–50%), hemicellulose (20–40%) and lignin (10–40%), and these weight concentrations vary depending on the plant material. In addition to these compounds, lignocellulosic biomass is also composed of inorganic material and extractives.^50,56^

Lignin and hemicellulose are removed by alkaline pretreatment and bleaching of cellulose fibers.^88^ Alkaline pretreatment uses strong bases such as NaOH, KOH, and Ca(OH)_2_, which break the bonds and cause swelling of the lignocellulosic material and, consequently, facilitate accessibility for cellulose extraction in the next steps.^56^ Bleaching, also called delignification, uses bleaching agents such as hydrogen peroxide (H_2_O_2_), sodium hypochlorite (NaClO) and sodium chlorite (NaClO_2_) to remove lignin.^89,90^

**Figure 5:**
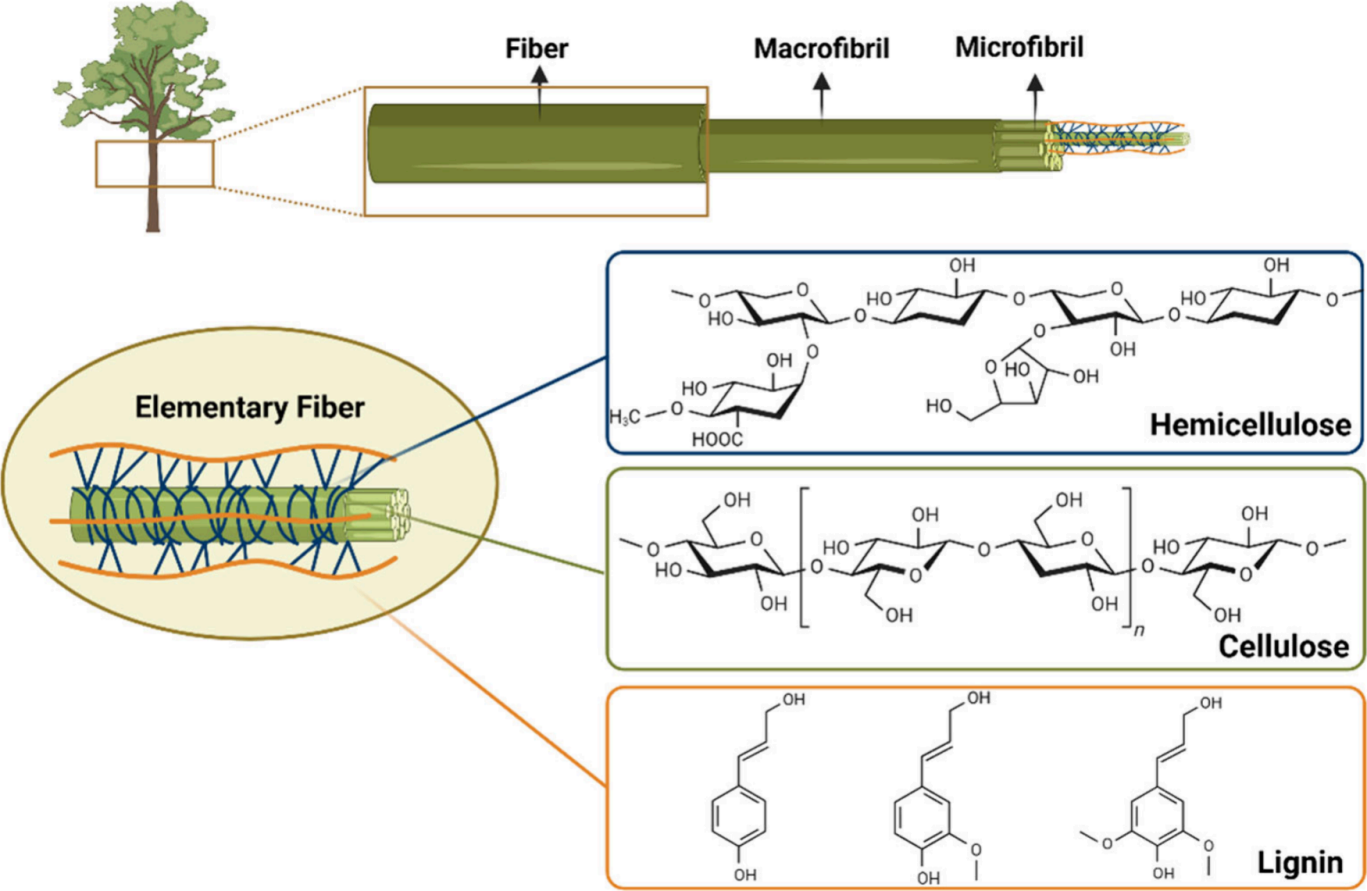
Representation of the structure of cellulose in plant biomass. Lignocellulose contains large amounts of lignin and hemicellulose. Created with BioRender.com.

Cellulose is an innovative material due to its unique physicochemical characteristics, such as renewability, high strength, easy access and low cost.^33^ Its chemical structure is rich in hydroxyl groups that form intermolecular and intramolecular hydrogen bonding interactions, resulting in a highly ordered structure. This structure is present in plant fibers in the form of microfibrils, which, in turn, are formed by elementary fibrils, which represent the smallest morphological units of the fiber (Figure 5).^91^

**Figure 6:**
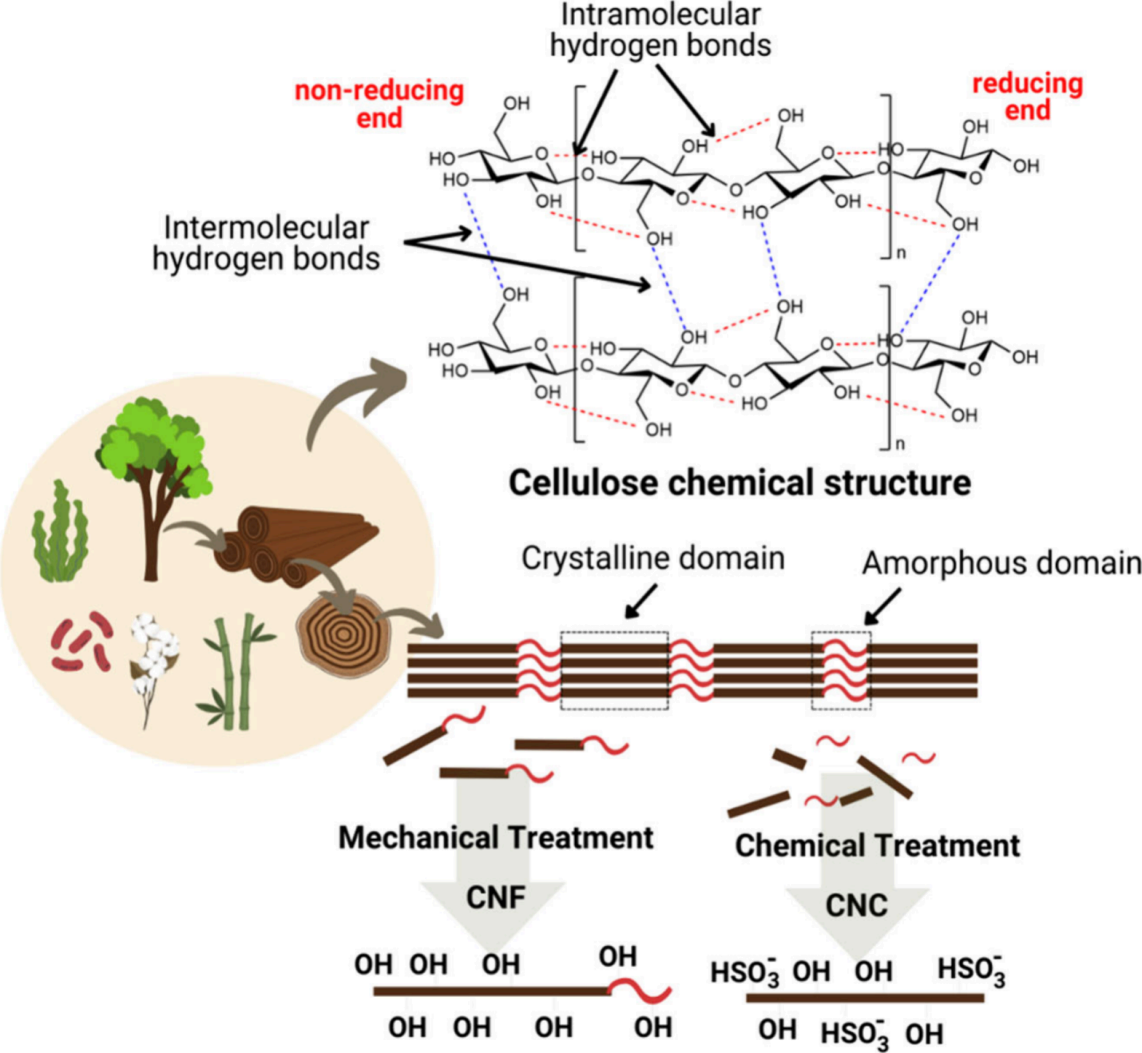
Molecular structure of cellulose and schematic representation of the production of CNCs and CNFs from wood pulp with their morphological and chemical structures. Created with BioRender.com.

These fibers have crystalline and amorphous regions, and the amorphous regions are degraded by mechanical or chemical processes, releasing the components of the crystalline region on the nanoscale. Cellulose is a dimer of d-glucose, called cellobiose, with linear units of β-d-glucopyranose linked by β-1,4 bonds. The terminal groups confer directional asymmetry to the molecule; one reducing end contains a hemiacetal group, and the other nonreducing end contains a hydroxyl group (Figure 6).^92^

The chemical structure of cellulose has β-d-glucopyranose rings with a ^4^C_1_ chair conformation, where the hydroxyl groups are in the equatorial plane and the hydrogens are in the vertical axial position. The intramolecular hydrogen bonds that stabilize cellulose occur between the C_3_ of one glucose unit and the O_5_ of the pyranose ring of another glucose unit and between the hydroxyls of C_6_ and C_2_′. These linkages result in three different positions of the hydroxymethyl groups: (1) *gauche–trans* (gt) when O_6_–C_6_ is *gauche* to O_5_–C_5_ and *trans* to C_4_–C_5_; (2) *gauche–gauche* (gg) when O_6_–C_6_ is *gauche* to O_5_–C_5_ and *gauche* to C_4_–C_5_; and (3) *trans*-*gauche* (tg) when O_6_–C_6_ is *trans* to O_5_–C_5_ and *trans* to C_4_–C_5_. The angle of twist (ω) in the *gauche conformation* is 30° < |ω| < 150°, and |ω| > 150° for the *trans* configuration. The crystalline surface of cellulose is preferably formed by gt or gg conformations.^61,93^

Nanocellulose (NC, a term used for nanostructured materials based on cellulose) is a particle that efficiently stabilizes the oil–water interface and meets the demand for a green and sustainable stabilizer for Pickering emulsions.^83^ NCs are derived mainly from cellulosic substrates from plant materials or bacterial sources, have at least one dimension at the nanometer scale (<100 nm) and are divided into three types: cellulose nanocrystals (CNCs), cellulose nanofibers (CNFs) and nanocellulose bacteria (BNC).^94^

**Figure 7:**
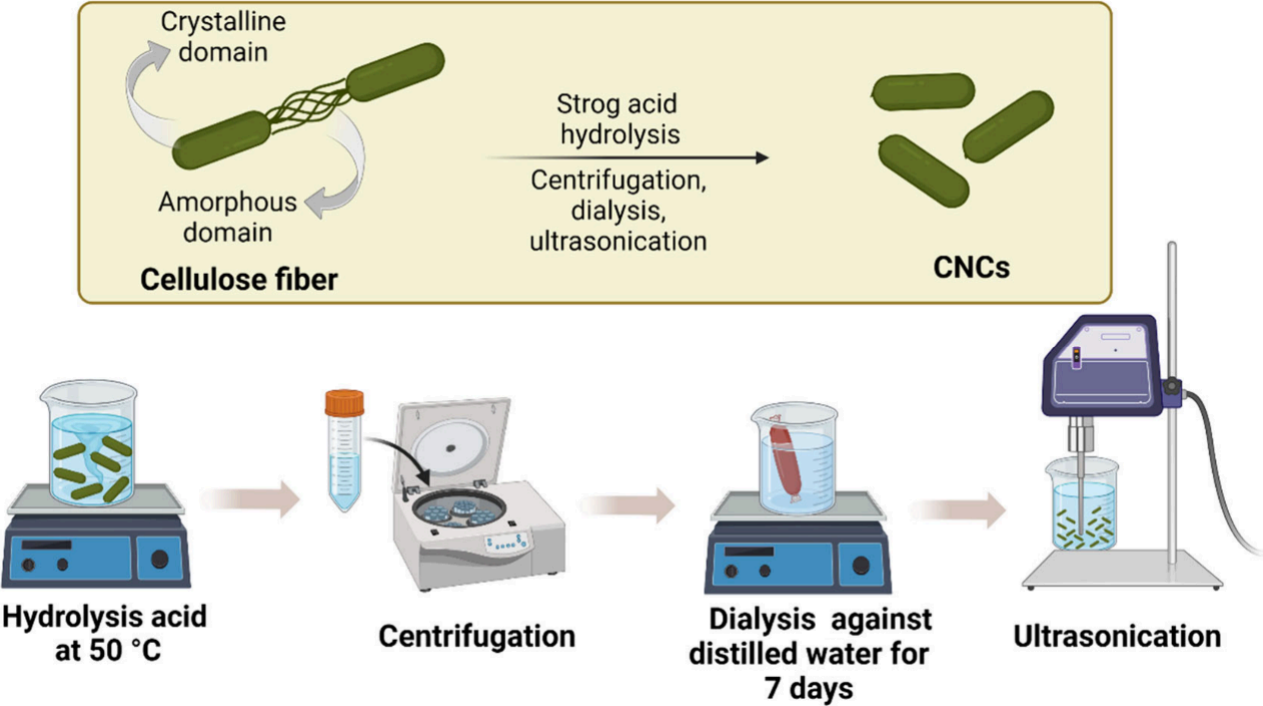
Schematic representation of CNC extraction steps from cellulose fibers through acid-catalyzed hydrolysis. The amorphous regions are hydrolyzed in an acidic medium, whereas the crystalline regions remain with the structure intact, producing CNCs with high crystallinity and a rod-shaped morphology. Adapted with permission from *Prog. Polym. Sci.***2021**, 119. 10.1016/j.progpolymsci.2021.101418.^34^ Copyright 2021, Elsevier. Created with BioRender.com.

CNCs (also known as cellulose nanowhiskers) are stick shaped and are formed from the crystalline region of cellulose, and the amorphous region is selectively removed by chemical treatment.^95^ Their dimensions range from 50 to 500 nm in length and from 5 to 10 nm in width. CNCs were initially produced by Rånby^96^ by dispersing cellulose fibers in water under acid hydrolysis. In chemical treatment, acid hydrolysis is used, which cleaves the glycosidic bonds of the amorphous region, which are easily accessed by the acid due to the disorder of the microfibrils, releasing crystalline fragments. The crystalline regions are highly ordered and, therefore, resistant to acid attack and remain intact after hydrolysis. The hydrolysis process is followed by centrifugation and dialysis to obtain a neutral pH in the solution, which then passes through an ultrasound probe to disperse the CNCs (Figure 7).^34,97^

The size and physical–chemical and surface parameters of CNCs influence their macroscopic properties, such as their rheology, morphology, dimensions, and colloidal stability. Various acids have been reported for the hydrolysis process, including sulfuric, hydrochloric, hydrobromic, and phosphoric acids.^95^ The type and concentration of acid used influence the dispersion and stability of the CNCs. For example, in sulfuric acid-catalyzed hydrolysis, anionic sulfate groups (OSO^–^_3_) are inserted on the surface of CNCs, resulting in electrostatic colloidal stability and easy dispersion in aqueous media. In comparison, hydrolysis catalyzed by hydrochloric acid produces weakly charged CNCs that tend to flocculate due to their low charge density.^98^

Nanofibrillated cellulose (CNF) is formed by filaments that comprise both the amorphous and crystalline regions.^95^ Initially, Turbak et al.^99^ obtained CNFs by passing a wood pulp suspension through a high-pressure homogenizer and proposed it as a cellulosic material with lateral dimensions in the nanometric range. CNFs exist in the form of clusters of fibrils with dimensions characterized by diameters on the manometric scale, usually ranging from 1 to 100 nm, and lengths on the micrometer scale. CNFs have unique characteristics, such as good mechanical properties, a high surface-to-volume ratio and the possibility of forming highly porous meshes.^100^

**Figure 8:**
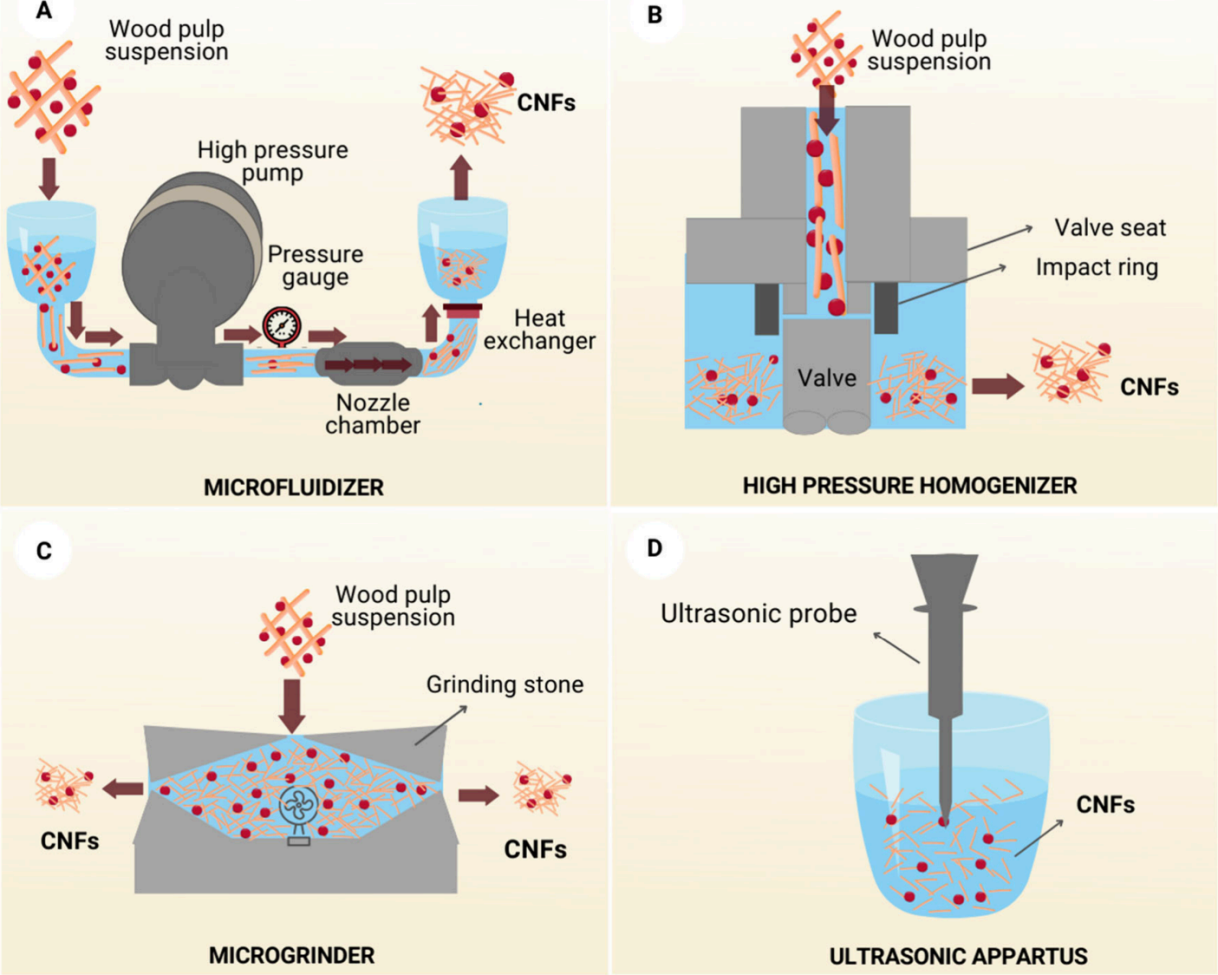
Schematic illustration of typical microfluidization (A), high-pressure homogenization (B), microgrinding (C), and high-intensity ultrasound (D) processes used to produce NFCs. Adapted with permission from *Prog. Polym. Sci.***2021**, *119*. 10.1016/j.progpolymsci.2021.101418.^34^ Copyright 2021, Elsevier.

For CNF extraction, cellulose fibers are commonly subjected to high-shear mechanical treatment, such as high-pressure homogenization,^99^ microfluidization,^101^ grinding,^102^ electrospinning,^103^ extrusion^104^ and high-intensity ultrasound,^105^ some of which are illustrated in Figure 8. The force applied to cellulose fibers during mechanical treatment determines the size and properties of the CNFs. The limitation of mechanical treatment is the high energy consumption (∼20,000–30,000 kWh/ton pulp) for fibrillation during fiber delamination. Therefore, chemical or enzymatic pretreatments are commonly combined with mechanical treatment to reduce the consumption of cellulose to <1,000 kWh/ton.^106^ A comprehensive description of mechanical treatment techniques is beyond the scope of this review. The interested reader is kindly directed to the scientific literature on this topic.^107−109^

Compared with conventional NPs, cellulose-derived NPs have advantages such as high crystallinity and strength, low density, and good modifiability. These compounds are hydrophilic and disperse well in strongly polar solvents, mainly in water, due to the abundance of hydroxyl groups on the surface of nanocellulose.^110^ However, in oil reservoirs, the dispersion of NCs may present low stability due to the high-salinity and high-temperature conditions found in this environment. The mono- and bivalent ions present in the formation water of reservoirs can cause aggregation and precipitation of NCs. This low stability could damage the reservoir and limit the application of NCs in EOR projects.^111,112^ In view of this, researchers have sought to develop methods for modifying the surface of nanocellulose to improve its stability in the face of adverse conditions in reservoirs. From this perspective, the advantage of NCs is that the presence of hydroxyl groups allows easy chemical modification of their surface, which can increase their hydrophobicity and adjust their interfacial behavior.^113^

### 3.1 Modification of the Surface of Nanocellulose

**Figure 9:**
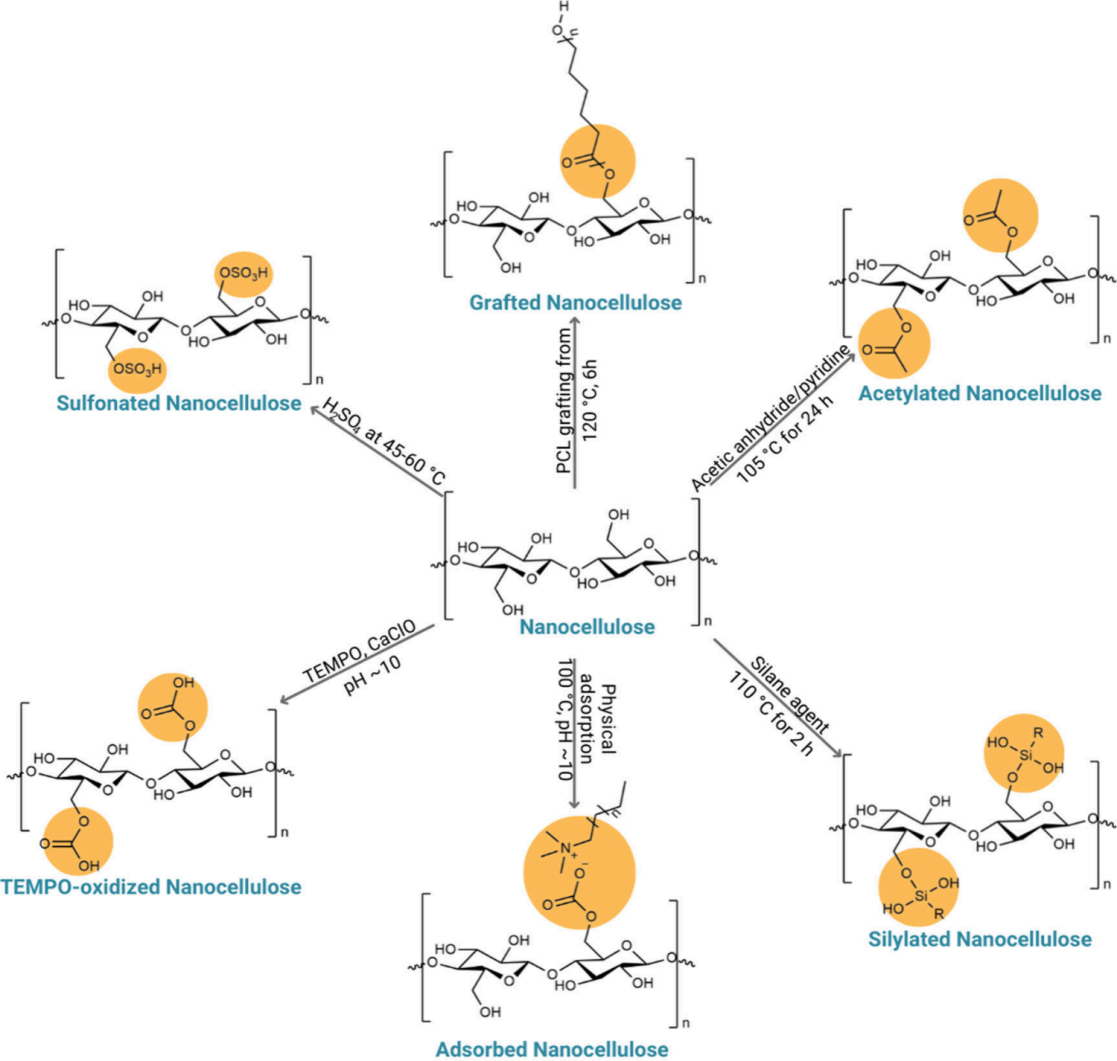
Schematic representation of the main surface modification techniques applied to cellulose NPs. The functional groups introduced are highlighted in orange. Adapted with permission from *Sci. Total Environ.***2021**, *775*, and 145871. 10.1016/J.SCITOTENV.2021.145871.^106^ Copyright 2021, Elsevier.

Cellulose NPs have been applied in several fields; however, their highly hydrophilic surface limits their application. To change the surface characteristics, chemical and/or physical modifications are used to adjust the hydrophilic–hydrophobic balance and expand the range of applications of NCs in other fields (Figure 9).^114^ Unmodified nanocellulose stabilizes only O/W emulsions. To stabilize W/O emulsions, hydrophobic modifications are required.^71^

Several routes described in the literature are used to modify the surface of the NC.^115^ Commonly, covalent bonds of hydrophobic molecules to hydroxyl groups on the surface of NCs are formed by oxidation,^116^ siliation,^117^ sulfonation,^118^ and acetylation,^119^ among other processes (Figure 9).

#### 3.1.1 TEMPO-Mediated Oxidation

The tetraalkylnitroxyl TEMPO radical (2,2,6,6-tetramethylpiperidine-1-oxyl) has been well described as an efficient oxidation catalyst for industrial application.^120^ During oxidation, high-density negative charges are inserted on the surface of the NCs, which promotes increased stability in aqueous suspensions. The reaction occurs in the presence of sodium hypochlorite (NaClO) and sodium bromide (NaBr), which selectively react with the hydroxyl group at C6 to form a carboxylate group.^121^ Although TEMPO-mediated oxidation has been used since 1966, Davis and Flitsch^122^ initially demonstrated its application in carbohydrates by reacting primary alcohols of carbohydrate glycosides using a biphasic dichloromethane/water system in the presence of TEMPO/NaClO/NaBr.

According to several methodologies, the ideal conditions for the TEMPO oxidation system of NCs are in water and in the presence of TEMPO/NaClO/NaBr at a pH of ∼10. The basic pH is fundamental for primary hydroxyls to react selectively, preventing the formation of aldehydes.^34^ TEMPO is a cyclic secondary amine nitrogen oxide with an unpaired electron between the nitrogen and oxygen atoms called the nitroxyl radical. This radical is a species generated by electron transfer and is present in a series of redox compounds, such as nitrosonic ions, hydroxylamine and TEMPO. Hydroxylamine reacts with nitrosonium ions to regenerate TEMPO, which is then continuously regenerated by NaClO, which is a primary oxidant.^116,121^

Isogai and Bergström^116^ described TEMPO-mediated oxidation as selective only for C_6_-hydroxy groups on the surface of crystalline cellulose microfibrils. In addition, the crystalline morphology was preserved, and the procedure fit within the concepts of green chemistry under aqueous conditions at room temperature and atmospheric pressure, which is considered a sustainable process.

#### 3.1.2 Acetylation

Acetylation is a widely used esterification reaction to partially replace the hydroxyl groups of the anhydro glucopyranose units of cellulose chains with acetyl groups. This reaction commonly occurs when acetic anhydride is used in the presence of sulfuric or perchloric acid as a catalyst.^119^ The degree of acetylation depends on the morphology, accessibility, and reactivity parameters of the nanocellulose. There are three hydroxyl groups that can be substituted, and the degree of acetylation can be expressed as the degree of substitution: GS 1 when one hydroxyl group is acetylated, and GS 3 when all three hydroxyl groups are acetylated.^123^ Acetylation reduces the number of hydroxyl groups on the surface of the NC; consequently, its hydrophilicity also decreases and the NC becomes more hydrophobic. Nanocellulose with GS 1 disperses better in polar solvents, whereas those with GS closer to 3 disperse better in hydrophobic apolar solvents.^124^

Singh et al.^125^ subjected wheat straw CNFs to acetylation using propionic anhydride, which demonstrated better dimensional stability than acetic anhydride. The reaction catalyst was sulfuric acid, and pyridine was described as the best solvent for obtaining high GS, as it increases the number of accessible hydroxyl groups. The GS was 2.77, making the surface of the CNFs more hydrophobic, and the morphology and crystallinity were not affected.

#### 3.1.3 Silylation

The silylation reaction is another widely used route for NC surface modification in which silyl groups are inserted to reduce the hydrophilicity of the material and improve its dispersion in apolar solvents.^106^ During silylation, hydrolysis and condensation occur. Initially, the mechanism occurs through the hydrolysis of hydroxyl groups, which are replaced by alkoxy groups in the presence of a catalyst. Next, the alkoxysilane and silanol condense into Si-O-Si bridges, eliminating either alcohol or water. A subsequent esterification and depolymerization mechanism results in the formation of Si–OC bonds.^126^

A series of organofunctional silanes, such as hexamethyldisilazane (C_6_H_19_NSi_2_), N-(β-aminoethyl)-γ-aminopropyl-trimethoxysilane (C_8_H_22_N_2_O_3_Si), 3-aminopropyltriethoxysilane (H_2_N(CH_2_)_3_Si(OC_2_H_5_)_3_), 3-glycidoxypropyltrimethoxysilane (C_9_H_20_O_5_Si), triethoxyvinylsilane (C_8_H_18_O_3_Si), and trichloromethylsilane (CH_3_Cl_3_Si), have been applied in NC silylation reactions.^106,127−129^

Goussé et al.^130^ described a surface silylation reaction of CNFs and CNCs using isopropyl-dimethylchlorosilane in toluene. The authors reported that under mild silylation conditions (low volume of reagents and shorter reaction time, with a GS between 0.6–1), the NCs maintain their morphology and obtain good dispersion in organic solvents. When the silylation reaction is severe (GS > 1), partial solubilization and loss of structure occur. Furthermore, compared with CNCs, CNFs are more resistant to silylation conditions because they support a greater concentration of reagents without loss of morphological structure.^129^

Zhang et al.^131^ reported the synthesis of hydrophobic, ultralight and flexible CNFs via silylation in an aqueous medium to obtain sponges and remove oil from water. The procedure used methyltrimethoxysilane and was highly efficient and simple for obtaining high-porosity sponges (>99%). Compared to inorganic sponges, silylated CNF sponges demonstrated greater flexibility due to the presence of Si, which can recover approximately 95% of its original thickness after compression. In addition, the silylated sponges were selective in removing dodecane from water because they have hydrophobic/oleophilic characteristics and can be recycled.

#### 3.1.4 Sulfonation

In the sulfonation reaction, the hydroxyl groups on the surface of the NCs are replaced by negatively charged semi-ester sulfate groups. The presence of this negative charge reduces the hydrophilicity, suppresses hydrogen bonding by electrostatic repulsion, improves dispersion in aqueous media and results in stable colloidal suspensions.^106^ Sulfonation occurs via hydrolysis by sulfuric acid. Its sensitivity to reaction parameters such as time, temperature and acid concentration make it difficult to control the amount of semi-ester groups that will be grafted onto the surface of NCs.^132^

Luo et al.^118^ studied CNFs subjected to sulfonation and compared them with CNFs modified by TEMPO-mediated oxidation. The sulfonation reaction used chlorosulfonic acid, dimethylformamide (DMF) and a reaction time of only 5 min. The authors reported that the CNFs demonstrated significant improvements in dispersibility and that their morphological structure was not damaged. Sulfonated CNFs demonstrated better dispersion than oxidized TEMPO-CNFs. Naderi et al.^133^ synthesized sulfonated CNFs and compared their properties with those of carboxymethylated CNFs. The conditions for carboxymethylation were ethanol, sodium hydroxide and vinylsulfonate solution at 80 °C for 3 h, after which the suspension was neutralized with acetic acid. Thus, compared to carboxymethylated CNFs, sulfonated CNFs were more stable under different pH conditions and demonstrated better redispersion properties (>50%). These characteristics were attributed to the sulfate group, which has a lower p*K*_a_ value, is larger in size and serves as a base. However, sulfonation affected the crystallinity, causing a decrease from 61% to 41%.

#### 3.1.5 Polymer Grafting

Another strategy for modifying the surfaces of NCs is the grafting of polymers onto hydroxyl groups, which function as chemical loops. Suspensions obtained by grafting polymers are highly stable in apolar solvents, and their interfacial properties can be adjusted according to the nature of the polymer.^34^

The grafting mechanism is classified into two approaches: (i) “grafting from” and (ii) “grafting into”. In the first approach, the mechanism involves mixing the NCs with a monomer and an initiator to induce polymerization. In the second approach, the mechanism involves the formation of a covalent bond between a coupling agent and a polymer with a reactive terminal group on the surface of the NC. In this strategy, the grafting density is lower than that in “grafting from” due to steric hindrance between the polymeric chains.^114,134^

Paquet et al.^135^ used the “grafting from” mechanism to insert polycaprolactone (PCL) chains with different molecular weights on the surface of microcrystalline cellulose (MC). The authors performed the reaction in three steps using phenylisocyanate to block one end of PCL and PCL diisocyanate 2,4-toluene to connect the other end to a hydroxyl group on the MC surface. In addition, preswelling conditions were not used to ensure that the reaction would be limited to the MC surface. According to the authors, natural fibers are sensitive to moisture and water, as the absorption and release of water cause swelling or shrinkage, which weakens the interface and leads to premature mechanical failure. Furthermore, several polymeric matrices are hydrophobic, and the presence of moisture can impair efficient adhesion between the matrix and the fiber. Therefore, preventing preswelling preserves the integrity of the interface and limits surface reactions. From the contact angle measurements, a significant increase in the hydrophobicity of the material produced was observed.

#### 3.1.6 Surfactant Adsorption

The surface of nanocellulose can be noncovalently modified via physical adsorption of oppositely charged compounds, surfactants or polyelectrolytes.^136,137^ Adsorption is an inexpensive and ecological method in which it is not necessary to use organic solvents. Furthermore, this approach is one of the easiest ways to modify the NC surface and can involve hydrophilic affinity, hydrogen bonding, electrostatic attraction, or van der Waals forces. These types of interactions are not as strong as chemical bonds; therefore, they can be easily broken by high shear forces.^106^

The use of cationic surfactants, which have a positive charge, is more applicable for modifying the surface of NCs, which commonly have negative charges. In these cases, it is common for surfactants to group together and adsorb in the form of an aggregate instead of adsorbing as an individual molecule, forming the more stable structures of bilayers and hemimicelles.^115^

Syverud et al.^138^ for example, used NFCs previously prepared by oxidation via TEMPO and mechanical fibrillation and modified their surface by the adsorption of cetyltrimethylammonium bromide (CTAB), a cationic surfactant. The modified CNFs demonstrated greater hydrophobicity and tensile strength in comparison to the unmodified CNFs.

### 3.2 Application of NCs in Pickering Emulsions

CNCs and CNFs are prone to form O/W-type Pickering emulsions that exhibit high stability against coalescence.^139^ CNCs have amphiphilic characteristics, with a hydrophilic face and hydrophobic edge plane of the crystalline faces of cellulose, which allows the stabilization of emulsions.^80^ CNFs are long fibers that form tangled three-dimensional networks around oil droplets, functioning as a shield against coalescence.^140^ The surface chemistry, wettability, size and morphology of NCs directly influence their adsorption behavior at interfaces and emulsion stability.^80^

There is a consensus that negatively charged modified NCs better stabilize Pickering emulsions due to electrostatic repulsion, which usually involves the addition of salts for electrostatic screening.^141^ Yan et al.^142^ prepared CNCs with deprotonated carboxylic groups, which were hydrolyzed by sulfuric acid, followed by peroxide oxidation, and evaluated their physicochemical properties and emulsifying performances in Pickering emulsions. The modified CNCs formed more stable emulsions than the unmodified ones due to electrostatic repulsion, which inhibited the coalescing mechanism. Furthermore, the authors used NaCl to increase the ionic strength by up to 80 mM and observed an improvement in the emulsion stability. This can be explained by the electrostatic shielding of sodium (Na^+^), which acts as a counterion and promotes the adsorption of CNCs at the oil–water interface. However, if the charge density of CNCs is very high, their adsorption at the interface is hampered.^80^ Kalashnikova et al.^71^ reported that sulfonated CNCs with high surface charge density do not stabilize emulsions. The authors concluded that the strong affinity of the CNCs for the aqueous phase prevented their adsorption at the O/W interface.

As previously described, the high hydrophilicity of NCs is a limiting factor for Pickering emulsion formulations. Therefore, it is necessary to increase their hydrophobicity through chemical or physical modifications to improve their emulsification performance.^71^

Hu et al.^143^ synthesized anionic CNCs coated with the cationic surfactants didecyldimethylammonium bromide (DMAB) and cetyltrimethylammonium bromide (CTAB) by physical adsorption (electrostatic interactions), leading to the formation of hydrophobic CNCs. Increasing the hydrophobicity of CNCs by adding surfactants increased the stability, improved emulsification, decreased the droplet size, and controlled the internal phase of the Pickering emulsions. With increasing amounts of DMAB, which is the most hydrophobic surfactant, a phase inversion of the transition from the position of W/O to the position of W/O and back to the position of the O/W was observed. The phase inversion was initially caused by the hydrophobization of the CNCs, and then the second inversion occurred due to the high concentration of DMAB, which stabilized the system. This induced phase inversion behavior was not observed with the CTAB surfactant.

Another important parameter in the formulation of Pickering emulsions is the concentration of nanoparticles, which influences the average droplet size and, consequently, the stability of the emulsion.^74^ Liu et al.^144^ investigated the stability of Pickering emulsions using sulfated CNCs extracted from sisal fibers at different concentrations (0.02, 0.025, 0.05, 0.1, 0.2 and 0.5%). When a concentration of 0.02% was used, the emulsion separated into two phases, and when the concentration increased, the emulsion remained highly stable for several months. Stability is achieved through the adsorption of nanoparticles at the oil–water interface and interactions between the adsorbed nanoparticles, forming a three-dimensional network that reduces the system’s free energy. Therefore, increasing the concentration of CNCs resulted in a larger total interfacial area of CNCs and more easily formed stable Pickering emulsions, but it is important to note that there is a limit to this concentration, beyond which stability may be compromised.

## 4 Application of Nanocellulose and Pickering Emulsions in EOR Mechanisms

By modification of the surface of cellulose nanoparticles, their colloidal properties can be adjusted to obtain materials with excellent interfacial properties. As a result, cellulose NPs demonstrate great potential for application in oil and gas production and in EOR projects. Their nanoscale size has good injectivity in reservoirs of low permeability and porosity, in addition to modifying rheological behavior and stabilizing emulsions, due to the extensive network of hydrogen bonds.^145,146^ Furthermore, as cellulose NPs are biodegradable and recyclable, they can reuse water in accordance with environmental regulations, generating economic gains for the oil and gas sector.^6^

The behavior of NPs in the porous media of reservoir rocks has been widely investigated. However, oil recovery by NPs is still not fully understood, and further studies focusing on rheological behavior, rock adsorption, flow and interfacial activity are needed.^147^ The most common mechanisms in discussions of NPs in EOR are interfacial tension (IFT) reduction, wettability changes, structural disjoining pressure, crude oil emulsification, rheology control, pore channel obstruction, and the prevention of asphaltene precipitation. In this section, the literature on NC-based Pickering emulsions and their influence on the main enhanced oil recovery mechanisms will be reviewed.

### 4.1 IFT Reduction

IFT is the force that exists between molecules at the interface of two immiscible fluids. Reducing the IFT to ultralow values (from 30 to <10^–3^ mN/m) is one of the most important mechanisms in EOR projects to reduce residual oil retained in reservoirs.^148^ Capillary forces are responsible for retaining oil droplets in the pores of the rock, and such forces are a result of IFT, wettability and pore geometry.^149^ In addition, the reduction in IFT leads to the formation of Pickering microemulsions *in situ*, with resistance to flow in high-permeability zones, directing the flow to low-permeability zones and improving the crude oil extraction process.^150,151^

NCs need to adsorb in oil–water and form stable emulsions. Adsorption depends on (i) the kinetic adsorption barrier, (ii) the diffusion of nanoparticles to the interface, and (iii) the size and shape of the NPs. These parameters strongly depend on the chemical functional groups at the NCs surface. For this purpose, more hydrophobic surfaces or adsorbed surfactants are desired.^35,152^ NP adsorption studies are limited to uncharged spherical particles, and it is necessary to expand the knowledge about anisotropically charged particles such as NCs.^153,154^

Bergfreund et al.^155^ investigated the adsorption of needle-shaped anisotropic CNCs with an average length of 79 nm and sulfate ester surfaces using oils with varying polarities. The authors highlighted the need to evaluate the influence of the oil characteristics on the adsorption process. CNC adsorption was strongly dependent on the oil polarity, whereas the chain length did not significantly influence the polarity. CNC adsorption was limited to polar oils. Charge screening induced by the addition of 20 mM NaCl improved adsorption due to lower electrostatic repulsion and a reduced kinetic adsorption barrier.^156^

Compared with unmodified NCs, chemically modified hydrophobic NCs behave substantially differently at the oil–water interface. Hiranphinyophat et al.^157^ compared the IFT reduction between CNCs grafted with a PIIP polymer (poly[2-isopropoxy-2-oxo-1,3,2-dioxaphospholane]) and unmodified CNCs. The unmodified CNCs reduced the IFT by 10 mN/m at 0.4% by weight, whereas the graft-modified CNCs reduced the IFT by more than 30 mN/m at 0.1% by weight.

Li et al.^158^ investigated the surface grafting of nanocellulose with AMPS (2-acrylamido-2-methylpropanesulfonic acid) and aliphatic chains as hydrophobic groups (HGs) to create an environmentally friendly displacement agent for enhanced oil recovery (EOR). The modified nanocellulose significantly reduced the interfacial tension (IFT) between the oil and water phases to 1 × 10^–1^ mN/m. Additionally, the NC-based nanofluid achieved an increase of 6% in residual oil recovery. These findings show the potential of functionalized nanocellulose as an effective strategy for improving EOR efficiency.

The reduction in the IFT alone does not fully account for the efficiency of displacement achieved in enhanced oil recovery (EOR) processes. Instead, EOR additives modify the wettability and increase the structural disjoining pressure, which are properties that significantly contribute to EOR effectiveness and must be considered alongside IFT reduction.

### 4.2 Changes in Wettability and Structural Disjoining Pressure

Rock wettability is another important parameter because it influences the displacement and distribution of fluids in porous media.^159^ Wettability is defined by the tendency of a fluid to adhere to a rock surface in the presence of other immiscible fluids; that is, it is the relationship between the solid surface and fluid–fluid and solid–fluid interactions. Depending on the chemical composition of the rock, crude oil, and formation water, reservoir rocks can be oil-wet, water-wet, or have mixed wettability conditions. Changing the wettability of reservoir rock from oil wetting to water wetting provides greater oil recovery.^160^ The contact angle (θ) is used to define the wettability of a three-phase system. The surface of the reservoir rock is wetted with water if the water contact angle is <90° and wetted with oil if the water contact angle is >90°.^159,161^

**Figure 10:**
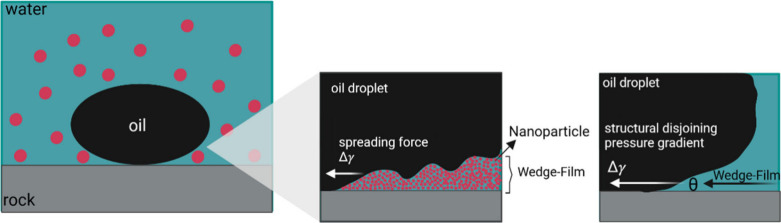
Schematic representation of the structuring of nanoparticles forming a wedge film, resulting in a structural disjoining pressure gradient. Adapted with permission from *Int. Nano Lett.***2019**, *9* (3). 10.1007/s40089-019-0272-8.^164^ Copyright 2019, Spring Nature. Created with BioRender.com.

To determine whether a surface is wetted by water or oil, interfacial and surface energies are used. The θ is calculated according to the balance between the scattering coefficient (*S*) of water on a solid surface in the presence of oil and that of water. NPs act by increasing *S* and removing the oil fraction from the solid surface.^162^ The scattering coefficient (*S*) of water on a solid surface in contact with water and oil is defined according to eq 9.^158^
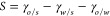
9where  represent the interfacial energies. NPs change the wettability of the rock by adsorbing on the rock surface, forming a wedge film, and the ordering of these NPs results in excess pressure (structural disjoining pressure) that displaces the oil droplets away from the rock surface (Figure 10).^163^

The diameter of the NPs is not large enough to counterbalance the other forces that act to separate them, such as electrostatic forces and van der Waals forces.^1^ Wedge film spreading is a function of the oil droplet volume and NP concentration. The speed of the internal contact increases as the NP concentration increases and decreases proportionally with a decreasing droplet volume. Altering the structural disjoining pressure mobilizes the oil retained in the pores of the rock at the microscopic level.^163^

Li et al.^158^ used NC modified by AMPS polymer grafting and hydrophobic groups (HGs) to alter the wettability of carbonate and sandstone rocks and compared their efficiency with that of unmodified NC. The wettability of oil-wet rock was successfully changed to water wetting due to the adsorption and scattering behavior of NC and NC-AMPS. Unmodified NC was more efficient in altering the wettability of sandstones, whereas modified NC was more efficient for carbonates. According to the authors, the reduction in the oil–water interfacial energy increases the scattering coefficient (S) and consequently reverts the surface to a water-wet state. The mechanism of the adsorption of NCs to rocky surfaces that alters the wettability is the result of electrostatic and van der Waals forces. The colloids spread out in the form of a wedge film and surround the oil droplet adsorbed on the rock surface, increasing the affinity of the surface for water.

Wei et al.^165^ synthesized nanofibrillated cellulose (NFC) grafted with AMPS and evaluated its efficiency in changing the wettability of sandstone rocks. The authors carried out studies of adsorption isotherms and images by scanning electron microscopy (SEM) and atomic force microscopy (AFM) and showed signs of the adsorption of NFC-AMPS on the rocky surface of sandstone. This adsorption behavior was explained on the basis of interactions such as hydrogen bonds, electrostatic forces, van der Waals forces, and hydrophobic interactions.

The ability to alter wettability is one of the fundamental mechanisms to explain, at the pore level, the increase in oil recovery for the fluids used. However, further studies are needed to correlate the adsorption, oil polarity, and surface charge of NCs and to understand the influence of the salinity and pH on these processes. In addition, other parameters need to be studied and correlated for a better understanding.

### 4.3 Crude Oil Emulsification

The *in situ* Pickering emulsion formation mechanism in reservoirs is a complex physical–chemical process, as it is influenced by several factors, such as rock wettability, pore structure, oil characteristics and distribution in porous media.^166^ Crude oil emulsification by Pickering emulsions is a highly desirable mechanism for the EOR processes and is related to interfacial activity. An ultralow IFT guarantees solubilization of the oil in the aqueous solution, forming a microemulsion, which facilitates the displacement of the oil in the porous medium.^164^

Crude oil emulsions stabilized by NPs have advantages over emulsions stabilized by surfactants. Their irreversible adsorption at the oil–water interface results in the formation of a film that protects the droplets against flocculation and coalescence, ensuring emulsion stability. NPs form kinetically controlled systems that maintain their morphology with increasing oil volume.^167^ Furthermore, due to their small size, stabilized Pickering emulsions can travel in porous media without retention or pore blockage problems. Finally, they are able to maintain their stability against adverse reservoir conditions such as high pressure, high temperature and high salinity.^168^

Raza and Gates^169^ used 2% CNCs by weight in a Hele–Shaw cell with narrow permeability and reported that CNC nanofluid droplets penetrate the oil phase and form stable Pickering microemulsions that result in greater oil displacement. Furthermore, this microemulsion effect further reduced the oil–water IFT. In conclusion, crude oil emulsification has proven to be one of the main EOR mechanisms for increasing the oil recovery efficiency.

### 4.4 Rheology Control

In EOR applications, determining the rheology of the fluid is essential for understanding its flow and deformation behavior in the face of stress in the reservoir and for determining ideal concentrations of NPs.^164^ Rheology is related to fluid injectivity and mobility in a porous medium. To obtain a favorable mobility ratio, the rheology of the fluid must be improved, that is, its viscosity increased by up to 100 cP, in a way that improves mobility without compromising injectivity.^2,165^

The rheological behavior of the fluid in the porous medium is given by the correlation between shear stress τ (force applied per unit area) and shear rate γ (shear per unit time). The ratio between the stress and shear rate therefore determines the viscosity (μ) of the fluid, which is defined by its resistance to flow.^147^

**Figure 11:**
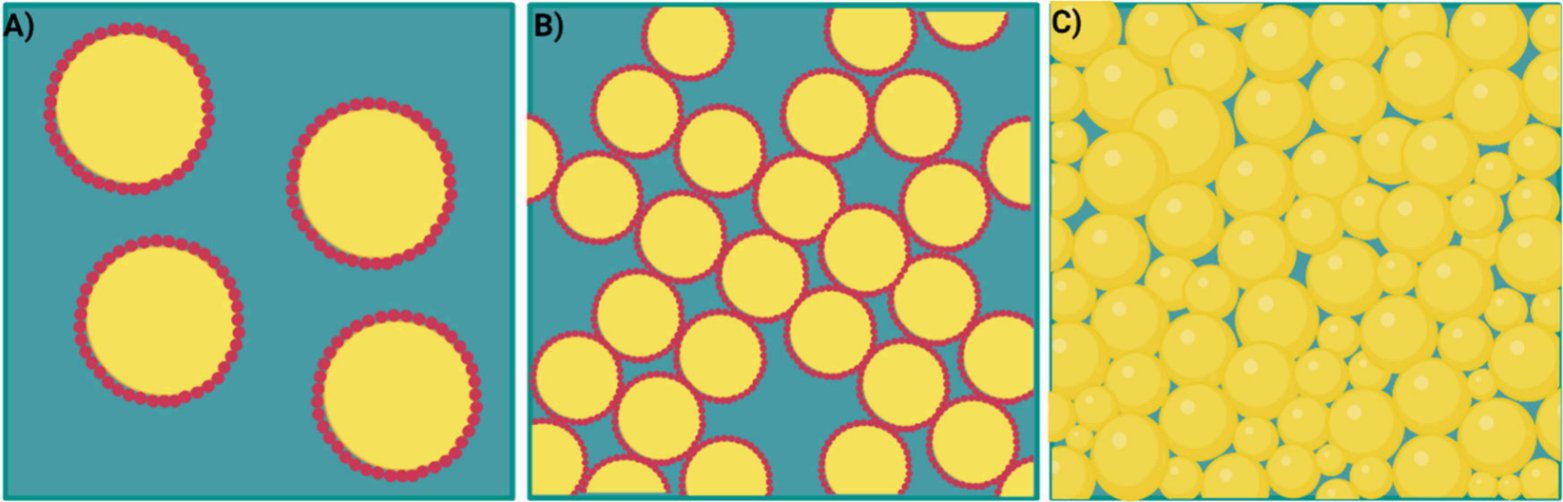
Scheme illustrating isolated droplets covered by colloidal particles at low volume fractions (a), medium volume fractions (b), and high volume fractions (c) of the dispersed phase. Created with BioRender.com.

Emulsions are composed of two phases and exhibit viscoelastic properties, where viscosity is related to friction within and between phases and elasticity arises from the energy stored in the deformed droplets.^64,170^ The rheology of emulsions is influenced by various factors, including interfacial tension (IFT), droplet size, viscosity of each phase, and volume fraction of the dispersed phase. Pickering emulsions can exhibit different rheological properties compared with conventional emulsions because of the rigid network structure in which particles form at the interface. At low volume fractions of the dispersed phase (Figure 11(a)), the energy stored in isolated droplets is easily dissipated in the continuous phase, resulting in a viscous response under shear stress or deformation. At medium volume fractions (Figure 11(b)) with particles of suitable wettability for the dispersed phase, two droplets can be adsorbed onto the same particle, forming a monolayer structure called a bridge. In this structure, the rheological behavior is more influenced by the rigid structure of the aggregated particles than by the IFT. At high volume fractions, where the volume of the dispersed phase exceeds 70%, emulsions known as HIPEs (high internal phase emulsions) (Figure 11(c)) present an extremely large interfacial area per volume. Consequently, significant elastic behavior is observed due to the high IFT. Thus, Pickering emulsions exhibit interesting rheological properties, as their behavior can be adjusted by altering parameters such as the volume fraction of the dispersed phase, particle volume fraction and particle type.^171,172^

CNCs and CNFs exhibit unique rheological behavior and are therefore employed in rheology control of various suspensions and emulsions.^173^ Li et al.^174^ compared the rheological behavior of CNFs and CNCs. The CNFs exhibited high viscosity, even at low concentrations, due to the formation of an entangled network, whereas the rheological behavior of the CNCs was significantly influenced by concentration. At low concentrations, the CNCs exhibited viscous, liquid-like rheological behavior, which shifted to elastic, gellike behavior as the concentration increased. Tanaka et al.^175^ evaluated the rheological behavior of different types of CNFs and CNCs obtained through TEMPO-mediated oxidation and reported that a higher aspect ratio leads to an increase in viscosity.

Wei et al.^165^ investigated the rheological behavior of O/W crude oil emulsions after the addition of CNFs and an alkaline solution. Commercial CNFs and another CNF associated with lignin were used. The two types of CNFs exhibited amphiphilic behavior and formed highly stable Pickering emulsions. Furthermore, as the viscosity increased from 1 cP to 10–30 cP, these values were within an adequate range to improve oil recovery, favoring injectivity and mobility. Emulsions with CNFs were more stable than emulsions with CNFs and lignin.

Wei et al.^176^ evaluated the influence of temperature on rheological behavior and compared it with that of hydrolyzed polyacrylamide (HPAM), the synthetic polymer most commonly used in EOR. When the compounds were subjected to temperatures of up to 100 °C, a slower loss of viscosity was observed for the CNFs than for the HPAM.

## 5 Conclusions and Future Prospects

Pickering emulsions stabilized by solid particles have attracted attention in the oil and gas industry. These particles have the ability to create a mechanical barrier around the droplets, irreversibly adsorbing at the interface. This capability of the particles is advantageous for providing greater stability to the emulsion and reducing the risk of coalescence and flocculation. Most of the research on cEOR has focused on Pickering emulsions with inorganic particles. However, there is a growing trend of using environmentally friendly compounds in cEOR. Cellulose nanoparticles are efficient materials for stable Pickering emulsion formulations. These biomaterials have attractive characteristics, such as high surface area, small size, the possibility of different active functional groups on the surface, and biodegradability. CNCs can be obtained from low-cost natural sources, primarily agricultural residues. The hydrophilic nature of CNCs results in low dispersibility in nonpolar solvents, which limits their application. The presence of hydroxyl groups on the surface of CNCs facilitates modification, adjusting their functional properties and providing sufficient hydrophobicity to expand their scope of application.

In the studies discussed here, Pickering emulsions based on CNCs acted through various mechanisms to increase the oil recovery rate. The reduction in interfacial tension is not significant for particle adsorption; what occurs is the formation of a mechanical barrier around the droplets, which prevents destabilization mechanisms. Overall, hydrophobically modified CNCs are more efficient in reducing IFT compared to unmodified CNCs. With the adsorption of CNCs at the interfacial region, oil solubilization occurs, forming an *in situ* microemulsion that facilitates their displacement in the porous medium. Due to the small size of the CNCs, the Pickering emulsions formed can travel through the porous medium without issues of retention and pore blocking. Additionally, CNCs alter the rock wettability by adsorbing onto its surface and forming a wedge film, which exerts a structural disjoining pressure that displaces the oil droplets from the rock surface. The choice of the chemical surface of CNCs can be altered according to the type of rock for a better efficiency. Overall, unmodified CNCs were more efficient at altering the wettability of sandstones, whereas modified CNCs were more efficient at altering the wettability of carbonates. Finally, rheological behavior is a fundamental mechanism for obtaining good injectivity and mobility of cEOR fluids in a porous medium. However, the high temperatures encountered in reservoirs lead to a loss of rheology in these fluids. Compared with a partially hydrolyzed polyacrylamide (HPAM), an emulsion using CNFs showed high temperature resistance.

This Review provides new perspectives and may identify potential opportunities for the implementation of new techniques aimed at increasing the oil recovery rate through the use of Pickering emulsions stabilized by CNCs. However, further studies are still needed to describe the behavior of these emulsions in a porous medium.
